# Novel structural protein in porcine reproductive and respiratory syndrome virus encoded by an alternative ORF5 present in all arteriviruses

**DOI:** 10.1099/vir.0.030213-0

**Published:** 2011-05

**Authors:** Craig R. Johnson, Theodor F. Griggs, Josephine Gnanandarajah, Michael P. Murtaugh

**Affiliations:** Department of Veterinary & Biomedical Sciences, University of Minnesota, St Paul, MN 55108, USA

## Abstract

Porcine reproductive and respiratory syndrome virus (PRRSV) is an arterivirus that emerged in the late 1980s in both Europe and North America as the causative agent of porcine reproductive and respiratory syndrome (PRRS), now the most important disease of swine worldwide. Despite extensive characterization of PRRSV proteins by direct analysis and comparison with other arteriviruses, determinants of virulence, pathogenesis and protective immune recognition remain poorly understood. Thus, we hypothesized that additional ORFs are present in the PRRSV genome that may contribute to its biological properties, and so we screened highly purified virions of strain VR2332, the prototype type 2 PRRSV, for evidence of novel polypeptides. A 51 aa polypeptide was discovered that is encoded by an alternative ORF of the subgenomic mRNA encoding the major envelope glycoprotein, GP5, and which is incorporated into virions. The protein, referred to as ORF5a protein, is expressed in infected cells, and pigs infected with PRRSV express anti-ORF5a protein antibodies. A similar ORF is present as an alternative reading frame in all PRRSV subgenomic RNA5 genes and in all other arteriviruses, suggesting that this ORF5a protein plays a significant role in arterivirology. Its discovery also provides a new potential target for immunological and pharmacological intervention in PRRS.

## Introduction

Porcine reproductive and respiratory syndrome virus (PRRSV) emerged in the late 1980s in both Europe and North America as the causative agent of porcine reproductive and respiratory syndrome (PRRS), characterized by late-term abortions and stillbirths in sows and interstitial pneumonia in nursery pigs (Collins *et al.*, 1992; Keffaber, 1989; Paton *et al.*, 1991; Wensvoort *et al.*, 1991). Now, PRRS has increased in virulence, causing widespread mortality in late-gestation sows and growing pigs of all ages; it is the most important disease of swine worldwide (Neumann *et al.*, 2005; Zhou & Yang, 2010).

PRRSV is an arterivirus, a family of positive-sense, single-stranded RNA viruses that includes: equine arteritis virus (EAV), first isolated in 1957 (Bryans *et al.*, 1957); lactate dehydrogenase elevating virus of mice, isolated in 1963 (Rowson *et al.*, 1963); and simian hemorrhagic fever virus, first identified in 1968 (Tauraso *et al.*, 1968). The arteriviral RNA is 5′ capped and 3′ polyadenylated with two large ORFs, 1a and 1b, which encode non-structural proteins, followed by structural protein ORFs at the 3′ end of the genome (Snijder & Meulenberg, 1998). Structural proteins are translated from a nested 3′-coterminal set of subgenomic mRNAs possessing a common leader derived from the 5′ end of the genome (Faaberg, 2008). An additional structural protein, E, is translated from an alternative ORF embedded within ORF2 (Wu *et al.*, 2001).

Despite extensive characterization of PRRSV structural and non-structural proteins by direct analysis, and comparison with other arteriviruses, the PRRSV determinants of virulence, cellular and tissue pathogenesis, and protective immune recognition remain poorly understood (Dokland, 2010; Faaberg, 2008; Snijder & Meulenberg, 1998). We hypothesized that additional ORFs are present in the PRRSV genome, whose protein products contribute to the biological properties of PRRSV. Since we were primarily interested in potential structural proteins, we screened highly purified virions for evidence of novel polypeptides. A novel 51 aa polypeptide was discovered by mass spectrometry. Surprisingly, it is encoded in an alternative reading frame of subgenomic mRNA 5 (sgmRNA5), which precedes the initiation site of the major envelope glycoprotein, GP5. The ORF, whose product is referred to here as ORF5a protein, is present as an alternative reading frame of sgmRNA5 in all PRRSV and all other arteriviruses, encoding a structurally similar putative membrane protein ranging in size from 43 to 64 aa.

## Results

### Identification of a novel structural protein

**Fig. 1.**
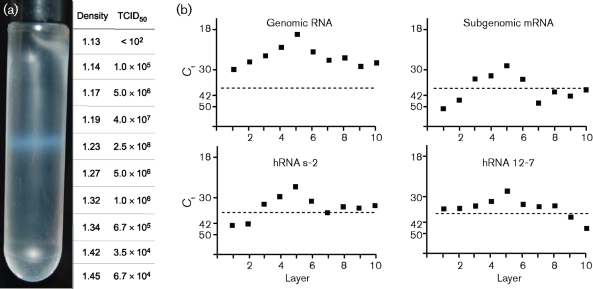
Physical characterization of PRRSV. (a) Banded PRRSV after centrifugation in CsCl. One millilitre fractions (layers) were collected, and numbered from the top of the gradient. Fraction densities and infectivities after dialysis are shown to the right. (b) Distribution of genomic, subgenomic and two heteroclite RNAs in the gradient. Dashed lines indicate the RT-PCR assay limit of detection (*C*_t_ = 38).

Initially, PRRSV virions were purified by commonly used methods involving rate-zonal centrifugation in sucrose (de Lima *et al.*, 2009; Yuan *et al.*, 2004). Liquid chromatography–tandem mass spectrometry (LC–MS/MS) revealed the presence of peptide sequences identical to the predicted tryptic peptides of a putative 51 aa protein encoded in sgmRNA5. However, the origin of the peptide could not be ascertained, since a wide range of non-structural proteins, indicative of cellular protein contamination, were also identified. Polyethylene glycol precipitation of clarified cell-culture supernatants at 48 h after PRRSV infection, followed by centrifugation through a sucrose cushion and isopycnic banding twice in caesium chloride (CsCl), resulted in the presence of a single virion population at a density of 1.22±0.1 g ml^−1^ (Fig. 1a). This band possessed the peak of biological infectivity, genomic RNA, subgenomic mRNA and heteroclite RNA (Fig. 1b). The abundance of subgenomic RNA and heteroclite mRNA was 100–1000-fold less than for genomic RNA, indicating that PRRSV assembles predominantly one type of infectious virion containing genomic RNA. Non-structural proteins were not present in any preparation, indicating that cellular proteins not specifically associated with virions had been removed quantitatively and that the purification procedure yielded viral particles in amounts sufficient for physical characterization.

**Fig. 2.**
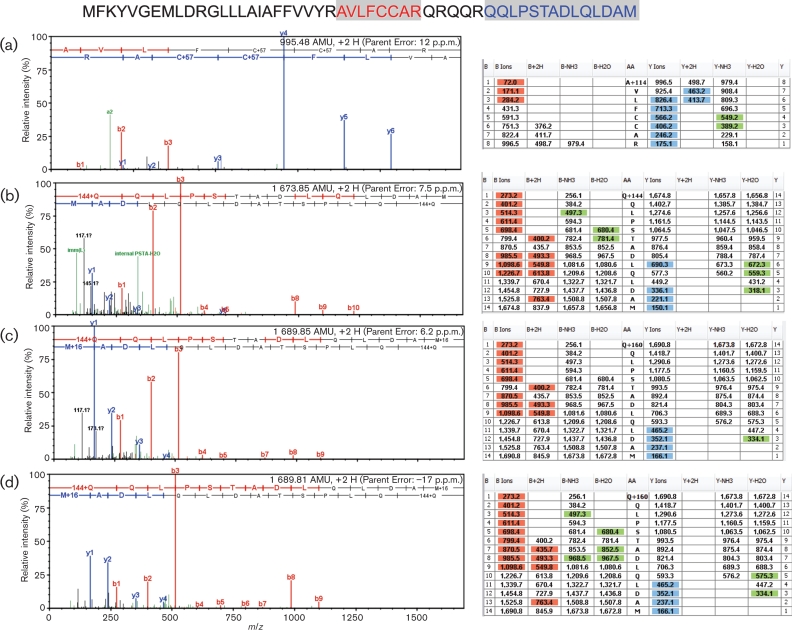
MS/MS sequencing of ORF5a-protein tryptic peptides. Peptide marked by red shading was identified in one sample (a). Peptide marked by blue shading was observed in three independent repetitions of the experiment (b–d). Spectra histogramas are shown to the left. Fragment ion tables are shown to the right. Amino acid sequences are in the column marked ‘AA’.

**Fig. 3.**
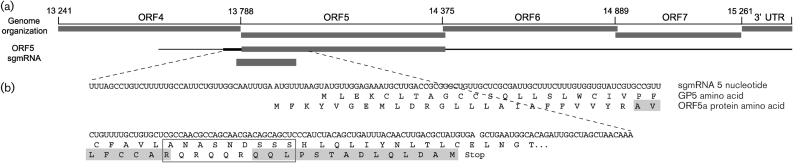
PRRSV strain VR2332 ORF5a protein. (a) Organization of the 3′ genomic region and subgenomic mRNA 5 (sgmRNA5) showing the ORF5a protein alternative reading frame. Initial thin line represents the leader sequence. Heavier line is the body sequence upstream of the AUG start site of ORF5. Thick line is ORF5. Downstream thin line represents the non-translated 3′ mRNA. Thick line below sgmRNA5 represents ORF5a. (b) Nucleotide sequence of sgmRNA5 showing the amino-terminal amino acid sequence of GP5 and the alternative ORF5a encoding the ORF5a protein. Shaded regions are the tryptic peptides identified by MS/MS sequencing. Boxed region shows the conserved R/Q-rich sequence.

**Fig. 4.**
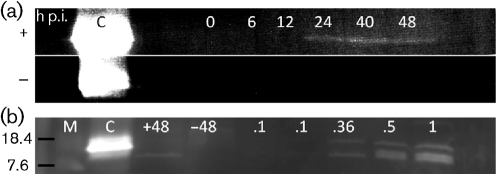
Immunoblot analysis of ORF5a protein. (a) Lysates of MARC 145 cells infected (+) or not infected (–) with PRRSV strain VR2332 were electrophoresed in an SDS-polyacrylamide gel and immunoblotted with goat anti-ORF5a protein antiserum. Specific anti-ORF5a protein staining is observed at time points after 20 h of infection; C, positive control sample of recombinant ORF5a protein, molecular mass 9.2 kDa. (b) ORF5a protein detection in purified virions by immunoblotting. Amount of viral protein loaded in lanes ranged from 0.1 to 1 µg as indicated. Lanes +48 and –48 represent lysates of cells incubated for 48 h with (+) or without (–) PRRSV. C, control recombinant ORF5a protein (9.2 kDa) as in (a). M, Kaleidoscope standards aprotinin (7.6 kDa) and lysozyme (18.4 kDa) (Bio-Rad).

iTRAQ (Applied Biosystems) analysis of four independently isolated virion preparations demonstrated, in every case, the same tryptic peptides that were previously observed in the crude virion preparation (Fig. 2). These tryptic peptides are encoded within a 156 nt alternative reading frame of sgmRNA5, hereafter referred to as ORF5a, and strongly suggest the presence of a novel 51 aa structural polypeptide in the virion. ORF5a precedes ORF5, which encodes GP5, by 10 nt (Fig. 3). Western blotting of ultrapurified virions with anti-ORF5a protein antisera raised in goat against the recombinant protein showed the presence of ORF5a protein migrating at the same rate as in infected cell lysates (Fig. 4). The protein migrated in the acrylamide gel with an apparent mobility of approximately 10 kDa, even though its predicted molecular mass is 5926 Da. Recombinant ORF5a protein with myc and histidine tags also migrates slowly relative to its molecular mass of 9228 Da (Fig. 4b). At higher virion protein loadings, ORF5a protein appears as a doublet and a slightly higher molecular mass band is also present, suggesting that ORF5a protein may be post-translationally modified.

### ORF5a protein expression in PRRSV-infected cells

**Fig. 5.**
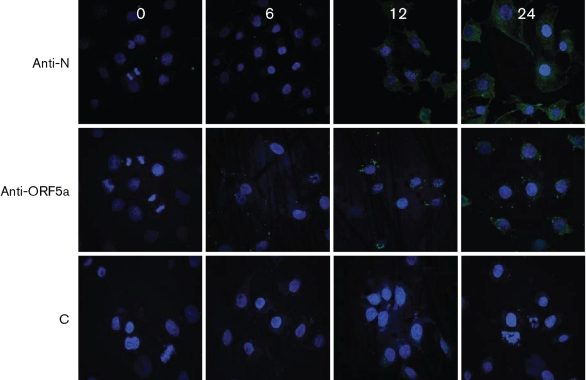
Immunofluorescent localization of ORF5a protein in PRRSV-infected MARC 145 cells. Cells were fixed at 0, 6, 12 and 24 h after infection (columns) and stained with fluorescein-labelled anti-nucleocapsid antibody (anti-N), goat anti-ORF5a protein antiserum immunodepleted to remove anti-myc antibodies (anti-ORF5a), and negative-control second antibody alone (C).

To gain more information about ORF5a protein, its kinetics of expression were determined following PRRSV infection of MARC-145 cells. As shown in Fig. 4, Western blotting revealed a faint chemiluminescent band at 24–48 h after infection. Immunofluorescent staining of infected cells showed sparse punctate staining at 6 h. At 12 and 24 h the staining was perinuclear, with or without a punctate cytoplasmic pattern at or near the plasma membrane (Fig. 5). The staining pattern was distinct from the cytoplasmic distribution of nucleocapsid (Fig. 5).

### Induction of anti-ORF5a protein antibody responses in pigs infected with PRRSV

**Fig. 6.**
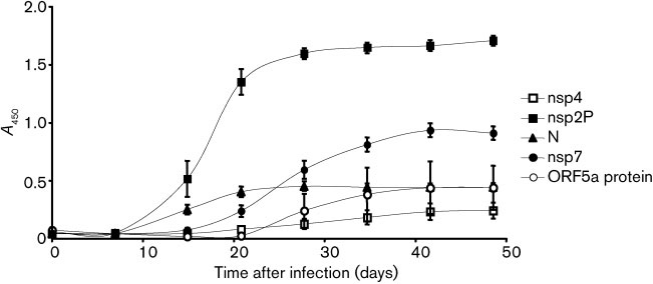
Kinetics of anti-ORF5a protein antibody response in pigs infected with PRRSV strain VR2332. Antibodies were first detected at 28 days after infection and reached a plateau at 42 days. Antibody responses to nsp4, nsp2 fragment P (nsp2P), nucleocapsid (N) and nsp7 also are shown for comparison. Data are mean±1 sem, *n* = 10 in all cases.

To determine if ORF5a protein was expressed during PRRSV infection of pigs, we tested for the presence of an anti-ORF5a-protein antibody response following infection. As shown in Fig. 6, specific antibodies were detected 4 weeks after infection and reached a plateau at about 7 weeks. The magnitude of the response was similar to that of anti-nsp4 antibodies, which also appear relatively late after infection and are a minor component of the anti-PRRSV humoral immune response compared with a range of other viral proteins.

## Discussion

### Discovery of a novel structural protein

**Fig. 7.**
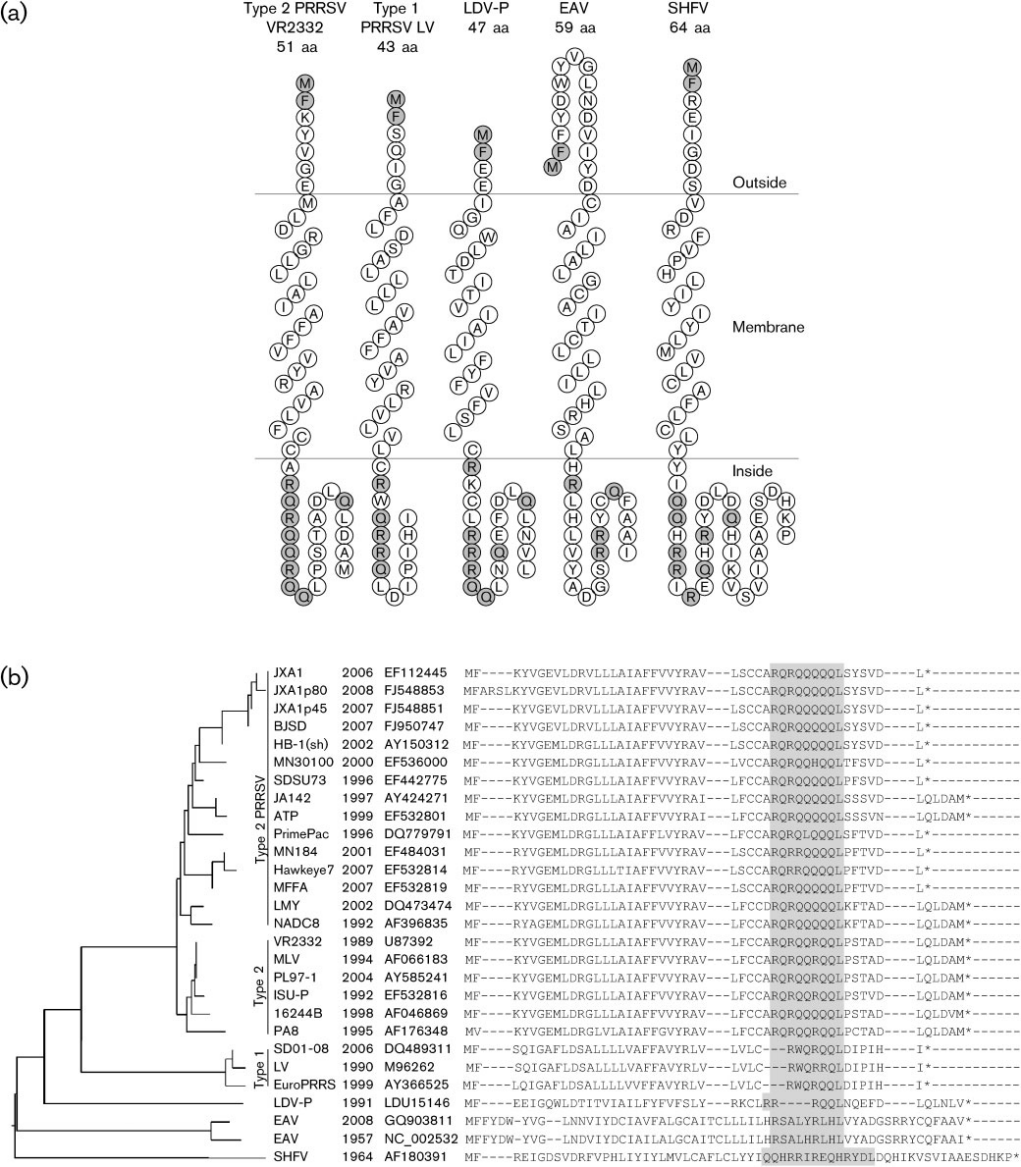
Comparison of ORF5a proteins in the *Arteriviridae*. (a) Topological prediction of ORF5a protein in type 1 and type 2 PRRSV, LDV, EAV and SHFV. Model predictions were performed in sosui (Hirokawa *et al.*, 1998). Conserved amino-terminal MF sequence and endodomain R and Q residues are highlighted in grey. (b) Phylogenetic analysis of ORF5a protein in the family *Arteriviridae*. Isolates are identified by common name, year of isolation, and GenBank accession number of the genome sequence. Major type 1 and type 2 PRRSV clusters are noted. Amino acid sequences were aligned in clustal x and a tree was constructed using neighbour joining and bootstrap analysis with 10 000 resamples. The tree was drawn in FigTree version 1.2.3. The shaded region shows the conserved R/Q-rich domain.

The 3′ terminus of the PRRSV genome, in addition to encoding known structural proteins, also contains approximately 20 potential polypeptide-encoding regions of 20 aa or more. Of these, peptides corresponding exactly to an alternative ORF encoding 51 aa in sgmRNA5 were present in several independently purified viral preparations. Cloning and sequencing of leader–body junctions of sgmRNA showed that the VR2332 sgmRNA5 would be expected to translate to GP5 or ORF5a protein (Nelsen *et al.*, 1999). The ORF5a protein initiator AUG codon is upstream of the consensus ORF5 AUG by 10 nt, with a second AUG representing a potential alternative ORF5a protein translation start site 11 nt downstream of the ORF5 start codon. The second AUG in the ORF5a protein is unlikely to initiate translation, since it is not conserved in PRRSV and is predicted to lie at the membrane surface (Fig. 7). Because the ORF5a protein initiator AUG appears first in sgmRNA5, ORF5a protein would be expected to be expressed at an appreciable level. The apparently low level of ORF5a protein expression suggests that ORF5, encoding GP5, is favoured as the start site of translation. The basis for this preferential expression of GP5 is not clear, since neither its initiator codon nor the two AUG sequences in ORF5b resemble the canonical Kozak sequence that typifies eukaryotic translational start sites (Kozak, 1987).

### ORF5a protein sequence analysis

PRRSV VR2332 ORF5a protein is predicted to be a type I membrane protein consisting primarily of alpha helix with a membrane-spanning domain (Fig. 7a) (Hirokawa *et al.*, 1998; Käll *et al.*, 2007; Rost *et al.*, 2004). There are two adjacent cysteine residues at positions 29 and 30, at the interior edge of the predicted transmembrane region (Fig. 7). They might allow for disulfide linkages to form in order to assist in oligomerization with other ORF5a protein monomers or with other proteins produced by the virus (Michelson, 2004; Wootton & Yoo, 2003). ORF5a protein has a highly conserved basic RQ-rich motif near the carboxyl terminus. Protein annotation by homology, domain analysis and secondary structure elements did not reveal features or motifs characteristic of known protein families (del Val *et al.*, 2007).

The C terminus of PRRSV ORF5a protein varies in length owing to the presence or absence of a 5 aa truncation, QLDAM, because of a cytosine→uracil transition that introduces a stop codon. Since the presence or absence of QLDAM is not correlated with any biological or phylogenetic property we are aware of, it might be dispensable to ORF5a protein function. An amino-terminal 4 aa insertion is present in cell culture passage 80 of a highly virulent Chinese PRRSV isolate, JXA-1, that is upstream of GP5 but maintains the ORF5a protein ORF, indicating that the length of the predicted ectodomain region may not be important to ORF5a protein function (Han *et al.*, 2009).

The ORF5a protein ORF is present in a wide variety of ancient and recent type 1 and type 2 PRRSV isolates (Fig. 7b). The phylogenetic organization into three clusters is consistent with the evolutionary divergence of type 1 and type 2 PRRSV, and the possible extinction of wild-type PRRSV isolates genetically related to strain VR2332, the prototype type 2 PRRSV (Shi *et al.*, 2010). The strain PL97-1, isolated in 2004, is, based on sequence similarity, most probably a field reisolate of the Ingelvac MLV vaccine. It is notable that the conserved RQ-motif of ORF5a protein is encoded by the same RNA sequence that encodes the hypervariable glycosylation-rich ectodomain region of GP5 (Fig. 3b, boxed region) (Kapur *et al.*, 1996). Interestingly, PRRSV contains another genomic region encoding ORF3, large parts of which also encode ORF2 and ORF4 (Meng *et al.*, 1995; Murtaugh *et al.*, 1995). This propensity for maintaining functionally intact, overlapping ORFs for essential viral structural proteins in the presence of high levels of nucleotide sequence diversity is an exceptional feature of PRRSV.

### Universal presence of the ORF5a protein ORF in PRRSV and arteriviruses

A predicted ORF5a protein highly similar to that encoded in the genome of VR2332 is present in all arteriviruses. The amino acid length varies from 46 to 51 in type 2 PRRSV, 43 in type 1 PRRSV, and from 47 to 64 in the other arteriviruses (Fig. 7). All of the predicted proteins contain an amino-terminal MF sequence, equivalent membrane topologies and R/Q-rich endodomain features (Fig. 7a). R and R/Q-rich motifs are involved in RNA binding that can regulate mRNA splicing and transport (Bayer *et al.*, 2005; Tan & Frankel, 1998), suggesting a possible role for the ORF5a protein. Mutational inactivation of ORF5a in EAV severely impaired replication, showing that maintenance of intact ORF5a is important for viability (Firth *et al.*, 2011). Thus, the ORF5a protein is not a happenstance sequence but represents a highly conserved ORF present throughout the family *Arteriviridae* whose function is not yet known, but may be related to efficient arteriviral RNA processing, transport or packaging.

The appearance of anti-ORF5a protein antibodies in swine following PRRSV infection shows that ORF5a protein expression is an intrinsic part of the PRRSV life cycle *in vivo*. The kinetics of antibody production show a slow appearance similar to that previously observed for anti-GP5 antibodies, and are consistent with the time course of neutralizing antibody production (Molina *et al.*, 2008; Mulupuri *et al.*, 2008). The low level of antibody expression is consistent with low levels of ORF5a protein synthesis and supports *in vitro* data indicating that it is present in low levels in infected cells and in virions. It is also possible that the ORF5a protein elicits a predominantly cell-mediated immune response rather than a humoral response (Jeong *et al.*, 2010).

Discovery of a novel structural protein, ORF5a protein, in PRRSV is supported by direct mass spectrometric evidence in purified virions, expression of the protein in cultured cells infected with PRRSV and induction of a specific immune response in infected pigs. A unique feature of the protein is its expression from an alternative ORF upstream of the highly expressed GP5 in sgmRNA5. It appears to be present in cells and virions at a low level, suggesting the presence of structural features in sgmRNA5 that do not favour translation from the first AUG. Its discovery provides a new potential target for immunological and pharmacological intervention in PRRS, the most important disease of swine worldwide.

## Methods

### 

#### Viruses.

The PRRSV isolate VR-2332 (GenBank number PRU87392) was propagated in MARC 145 cells maintained in EMEM medium (Invitrogen Life Technologies) containing 10 % FBS (Kim *et al.*, 1993). For large-scale preparation, cells in 75 cm^2^ flasks were infected at variable m.o.i. and incubated at 37 °C in a 5 % CO_2_ atmosphere. Infected cell supernatant was harvested at 48 h after infection, before a cytopathic effect was observed (Yuan *et al.*, 2001). Cellular debris was removed by centrifugation at 3800 ***g*** at 4 °C for 30 min. Supernatant was collected and polyethylene glycol 8000 (PEG 8000; Fisher Scientific) was added to a final concentration of 10 % (w/v) and stirred gently overnight at 4 °C. Precipitated proteins were collected by centrifugation at 17 700 ***g*** for 1 h at 4 °C. The pellet was resuspended in 50 mM HEPES, 100 mM NaCl, 1 mM EDTA, pH 7.5. The sample was carefully layered onto a cushion of 0.5 M sucrose in 10 mM NaCl, 10 mM Tris/HCl, 1 mM EDTA (STE), pH 7.5, in SW 32 Ti ultracentrifuge tubes (Beckman Coulter). Virus was pelleted at 110 000 ***g*** at 4 °C for 3 h. Pellets were collected, resuspended and pelleting was repeated once.

Pellets were resuspended in 1.25 g ml^−1^ CsCl, 50 mM HEPES, 100 mM NaCl, 1 mM EDTA at pH 7.5 and centrifuged in a type 70.1 Ti rotor at 247 000 ***g*** (Beckman Coulter) for 72 h. Viral bands and density-gradient aliquots were carefully extracted by syringe. Equilibrium density-gradient centrifugation was repeated one further time. Purified virions were dialysed against 50 mM HEPES, 100 mM NaCl, 1 mM EDTA, pH 7.5, at 4 °C with several buffer exchanges. CsCl layers were examined by refractive index (American Digital), quantitative RT-PCR (qRT-PCR) for viral RNA levels (Applied Biosystems) and TCID_50_ on MARC 145 cells.

#### Viral RNA isolation and qRT-PCR.

Viral RNA was isolated using a QIAamp Viral RNA Mini kit (Qiagen) or a Nucleospin II RNA Isolation kit (BD Biosciences), eluted into 50 µl of RNase-free water, and stored at −80 °C. Complementary DNA was synthesized with Superscript II reverse-transcriptase (Invitrogen Life Technologies). Primers shown in Supplementary Table S1 (available in JGV Online) were selected to span the appropriate region of the viral genome to identify specific populations of RNA, including heteroclite RNA, using primer3 software (Rozen & Skaletsky, 2000). Heteroclite RNA contains 5′ and 3′ genomic fragments with non-canonical leader–body junctions, similar to defective interfering RNA described in coronaviruses, but it does not interfere with growth (Yuan *et al.*, 2000). Primers were mixed with sample and 2× SYBR Green MasterMix (Perkin-Elmer Applied Biosystems). Quantitative RT-PCR was carried out with an ABI 7500 Sequence Detection System (Perkin-Elmer Applied Biosystems). Thermocycling conditions were 95 °C for 10 min, followed by 45 cycles of 95 °C for 15 s and 60 °C for 1 min (Baker *et al.*, 2007).

#### Expression of recombinant ORF5a protein and anti-ORF5a protein IgG production.

Recombinant ORF5a protein containing an amino terminal myc tag (MEQKLISEEDL) and a carboxyl terminal 6× histidine tag (LEHHHHHH) was cloned and expressed in *Escherichia coli* Rosetta 2(DE3) cells (Novagen) as previously described (Johnson *et al.*, 2007). Protein was purified using an Ni-NTA spin column (Qiagen) or HisPur Cobalt Resin (Pierce). ORF5a protein expression amounts and purity were monitored by SDS-PAGE. Purified recombinant ORF5a protein was sent to Bethyl Laboratories (Montgomery TX, USA) for antibody production in a goat.

#### Affinity purification of anti-ORF5a protein antibodies.

Recombinant ORF5a protein and control porcine haptoglobin α1S, also containing myc and histidine tags and expressed and purified in the same way as ORF5a protein, were immobilized on CNBr-activated Sepharose 4B (GE Healthcare) according to the manufacturer’s instructions (Gnanandarajah *et al.*, 2008; Johnson *et al.*, 2007). Affinity columns were stored at 4 °C in 20 % ethanol (v/v) after ligand coupling.

Anti-myc antibodies were removed on the haptoglobin α1S-Sepharose column, and anti-ORF5a-protein antibodies were purified from anti-myc-depleted serum on ORF5a protein–Sepharose. Sera were passed through a 0.45 µm filter and then passed through the affinity column by gravity flow twice. Removal or concentration of specific antibodies was monitored by ELISA as described by Johnson *et al.* (2007).

#### Western blot analysis.

Protein samples were electrophoresed in 10 % SDS-polyacrylamide gels and transferred to PVDF membranes (Immobilon-P; Millipore). Membranes were blocked in 5 % non-fat dry milk and 0.1 % Tween-20 (Sigma) in PBS (pH 7.4) overnight. Membranes were incubated with sera for 1 h at room temperature, washed, and incubated with HRP-labelled rabbit anti-goat IgG (KPL) at a 1 : 2000 dilution at room temperature. Enhanced chemiluminescence Western blotting detection reagents were used according to the manufacturer’s instructions (Thermo Fisher Scientific) to visualize the bands.

#### Immunofluorescent detection of ORF5a protein.

MARC-145 cells were grown for two days on glass coverslips and then infected with PRRSV strain VR2332 overnight. Coverslips were washed in PBS (pH 7.4), and cells were fixed in 80 % acetone, 20 % methanol at 4 °C for 20 min, followed by three washes in PBS. Coverslips were blocked in 1 % BSA in PBS for 1 h at 37 °C. Goat anti-ORF5a-protein antiserum depleted of anti-myc antibodies by affinity chromatography was diluted 1 : 1000 in 1 % BSA and centrifuged at 13 200 ***g***</bold> for 10 min to remove fine particulates, then applied to each coverslip for 1 h at 37 °C. Coverslips were washed 3 times in PBS and 100 µl of Alexa Fluor 488-labelled rabbit anti-goat IgG, diluted 1 : 2000 in 1 % BSA, was added for 45 min at 37 °C, followed by 3 washes in PBS. Fluorescein-labelled SDOW17 anti-nucleocapsid mAb (Rural Technologies) was used as a positive control. Cell nuclei were stained with 0.1 µg bisbenzimide ml^−1^ for 10 min at room temperature during the final wash. Excess moisture was blotted away and coverslips were mounted on clean glass slides. Immunofluorescence was observed by using an MRC 1024 laser confocal microscope (Nikon Diaphot Inverted; Bio-Rad) at 408 nm, 488 nm and 555 nm excitation frequencies.

#### Micro liquid chromatography–pulsed Q collision induced dissociation (µLC–PQD) MS/MS.

An automated Paradigm MS4 system (Michrochrom Bioresources) was used inline with an LTQ mass spectrometer (ThermoFisher) as previously described (Xie *et al.*, 2007). Buffer A was 0.1 % formic acid in a solution of 95 % acetonitrile and 5 % water. Peptides were eluted using a linear gradient of 10 to 35 % HPLC buffer B (0.1 % formic acid in a solution of aqueous 95 % acetonitrile) over 1 h followed by an isocratic elution at 80 % buffer B for 5 min (flow rate 0.25 ml min^−1^).

#### µLC-quadrapole time-of-flight (TOF) mass spectrometry.

An Online LC system (LC Packings/Dionex) was used on a QSTAR Pulsar i quadrapole-TOF MS instrument (ABI, Foster City, CA) equipped with a nanoelectrospray source (Protana, Denmark) (Kapphahn *et al.*, 2003). A qTOF MS electrospray ionization voltage of 1000 V was used, with a TOF region acceleration voltage of 4 kV and an injection pulse repetition rate of 6.0 kHz. Calibrations were performed using the [M+3H]^3+^ at 586.9830 mass/charge (*m*/*z*) monoisotopic peak and the [M+2H]^2+^ monoisotopic peak at 879.9705 *m*/*z* from human renin substrate tetradecapeptide (Sigma-Aldrich).

#### MALDI mass spectrometry.

Samples were desalted using a C18 ZipTip (Millipore). One microlitre of sample was combined with 1 µl of matrix dihydroxybenzoic acid (Agilent Technologies), spotted onto the target, and air-dried. Data were collected on a Biflex III MALDI-TOF mass spectrometer (Bruker Daltonics) operated in linear mode as described previously (Gnanandarajah *et al.*, 2008; Nelsestuen *et al.*, 2005) and an oMALDI-Qstar Pulsar i QqTOF mass spectrometer (ABI). Angiotensin II (monoisotopic mass [M+H]^+1^ 1046.5417 (Sigma-Aldrich) and adrenocorticotropic hormone (ACTH) fragment 18–39 (monoisotopic mass [M+H]^+1^ 2465.1989; Sigma Aldrich) were used for calibration. A nitrogen laser (337 nm, 33 µJ) with pulse repetition rate of 20 Hz was used and mean mass spectra were taken from 100 laser shots in positive mode.

#### LTQ-PQD data analysis.

The seaquest program (Bioworks version 3.2; Thermo Fisher) was used to search against a composite database consisting of all VR2332 ORFs >25 aa and a subset of monkey, pig and random contaminates from the NCBI (http://www.ncbi.nlm.nih.gov) non-redundant protein database of 15 May 2006. Search parameters included differential amino acid shifts for oxidized methionine (+16 Da) allowing for up to two partially missed internal tryptic peptides. Peptide tolerances were ±150 p.p.m. and MS/MS tolerance of ±0.40 Da was allowed.

#### QSTAR data analysis.

Protein Pilot version 1 (ABI) and mascot (Matrix Science) were used to analyse the data with the previously mentioned composite database. A generic set of variable amino acid modifications were used that included oxidized methionine and modifications for lysine, tyrosine and peptide amino-termini.
